# Soil Dust Aerosols and Wind as Predictors of Seasonal Meningitis Incidence in Niger

**DOI:** 10.1289/ehp.1306640

**Published:** 2014-03-17

**Authors:** Carlos Pérez García-Pando, Michelle C. Stanton, Peter J. Diggle, Sylwia Trzaska, Ron L. Miller, Jan P. Perlwitz, José M. Baldasano, Emilio Cuevas, Pietro Ceccato, Pascal Yaka, Madeleine C. Thomson

**Affiliations:** 1 NASA Goddard Institute for Space Studies, New York, New York, USA; 2 Department of Applied Physics and Applied Math, Columbia University, New York, New York, USA; 3 Lancaster Medical School, Lancaster University, Lancaster, United Kingdom; 4 Liverpool School of Tropical Medicine, Liverpool, United Kingdom; 5 Department of Epidemiology and Population Health, University of Liverpool, Liverpool, United Kingdom; 6 International Research Institute for Climate and Society, Palisades, New York, USA; 7 Barcelona Supercomputing Center–Centro Nacional de Supercomputación, Barcelona, Spain; 8 Izaña Atmospheric Research Center, Agencia Estatal de Meteorología, Tenerife, Spain; 9 Office of Civil Aviation and Meteorology General Direction, Ouagadougou, Burkina Faso; 10 Department of Environmental Health Sciences, Mailman School of Public Health, Columbia University, New York, New York, USA; * These authors contributed equally.

## Abstract

Background: Epidemics of meningococcal meningitis are concentrated in sub-Saharan Africa during the dry season, a period when the region is affected by the Harmattan, a dry and dusty northeasterly trade wind blowing from the Sahara into the Gulf of Guinea.

Objectives: We examined the potential of climate-based statistical forecasting models to predict seasonal incidence of meningitis in Niger at both the national and district levels.

Data and methods: We used time series of meningitis incidence from 1986 through 2006 for 38 districts in Niger. We tested models based on data that would be readily available in an operational framework, such as climate and dust, population, and the incidence of early cases before the onset of the meningitis season in January–May. Incidence was used as a proxy for immunological state, susceptibility, and carriage in the population. We compared a range of negative binomial generalized linear models fitted to the meningitis data.

Results: At the national level, a model using early incidence in December and averaged November–December zonal wind provided the best fit (pseudo-*R*^2^ = 0.57), with zonal wind having the greatest impact. A model with surface dust concentration as a predictive variable performed indistinguishably well. At the district level, the best spatiotemporal model included zonal wind, dust concentration, early incidence in December, and population density (pseudo-*R*^2^ = 0.41).

Conclusions: We showed that wind and dust information and incidence in the early dry season predict part of the year-to-year variability of the seasonal incidence of meningitis at both national and district levels in Niger. Models of this form could provide an early-season alert that wind, dust, and other conditions are potentially conducive to an epidemic.

Citation: Pérez García-Pando C, Stanton MC, Diggle PJ, Trzaska S, Miller RL, Perlwitz JP, Baldasano JM, Cuevas E, Ceccato P, Yaka P, Thomson MC. 2014. Soil dust aerosols and wind as predictors of seasonal meningitis incidence in Niger. Environ Health Perspect 122:679–686; http://dx.doi.org/10.1289/ehp.1306640

## Introduction

The meningitis belt in sub-Saharan Africa is the region where most epidemics of meningococcal meningitis occur and which suffers the greatest burden of endemic disease (Molesworth et al. 2002). Meningitis is an infection of the thin lining that surrounds the brain and spinal cord. Although there are many causes of meningitis, the epidemic form of the disease is caused by the bacterium *Neisseria meningitidis*. Human carriers transmit these bacteria through respiratory droplets or throat secretions. Under certain circumstances the bacteria become pathogenic, invading the nasopharyngeal epithelial cells and entering the blood stream, thus instigating disease. Epidemics in the meningitis belt are caused by serogroups A, C, X, and W135, with serogroup A meningococcus accounting for an estimated 80–85% of all cases.

Epidemic control and response has been based on reactive mass vaccination with meningococcal polysaccharide (PS) vaccines and effective case management. The deployment of PS is usually based on early detection of epidemics through the effective application of alert and epidemic thresholds as recommended by the World Health Organization (WHO 2000). Consequently, the impact of the vaccination response depends largely on the quality and timeliness of the surveillance system, and would benefit from forecasting tools.

Epidemics and seasonal upsurges in endemic disease occur in the latter part of the dry season after the onset of the Harmattan—a ground-level stream of dry and dusty desert air, which is part of the African continental trade wind system that sweeps southwestward between the end of November and the middle of March—and usually subside at the onset of the rains (Lapeysonnie 1963; Molesworth et al. 2002). The location and seasonality of meningitis epidemics suggest that environmental factors, such as low absolute and relative humidity, high temperatures, and dusty atmospheric conditions may play an important role (e.g., Agier et al. 2013; Cheesbrough et al. 1995; Dukić et al. 2012; Martiny and Chiapello 2013; Sultan et al. 2005). It also has been suggested that climate conditions may contribute to the year-to-year variation in the incidence of meningitis in specific locations (Thomson et al. 2006; Yaka et al. 2008).

The mechanism by which climate and dust may influence meningitis occurrence along with epidemic location and intensity remains unclear. The mechanisms of the interaction between *N. meningitidis* and the mucosal epithelial cells are well known (van Deuren et al. 2000), but, to our knowledge, there are no *in vivo* studies of the effects of climate and dust on the pathogenesis and transmission of *N. meningitidis* (Palmgren 2009). The most common proposed mechanism has been that physical damage to the epithelial cells lining the nose and throat in dry and dusty conditions permits easy passage of the bacteria into the blood stream, causing invasive disease. Other more controversial mechanisms involve potential effects of dust particles on the fluid dynamics of airborne bacteria transmission, the potential impact of climate (high dust levels, low humidity, and cold nights) on preceding viral infections that may increase susceptibility, effects of iron in dust particles on the activation of the meningococci, and effects of high dust levels on human behavior, including crowding and reduced ventilation (e.g., blocking windows).

Factors other than climate conditions, such as herd (i.e., population) and natural immunity levels, vaccination type and coverage, serogroup type, new strains, clonal virulence, and coincident respiratory infections, are likely to contribute to the temporal and spatial variation in meningitis epidemics (e.g., Mueller and Gessner 2010). However, despite progress in surveillance and research, efforts to predict epidemics have been hindered by an incomplete understanding of meningitis epidemic patterns and a lack of data (Moore 1992).

In this study we extended the work of Yaka et al. (2008) at the national level in Niger by testing seasonal forecast models based on climate and dust information, along with other determinants at both national and district levels, using data that would be readily available in an operational framework. We analyzed and compared a range of negative binomial generalized linear models that were fitted to the meningitis data.

## Data and Methods

*Epidemiological data: early and seasonal cases*. We used the number of weekly suspected cases compiled by the Multidisease Surveillance Center (MDSC) based on information provided by the Ministry of Public Health in Niger for 38 districts as defined before 2002. The period used in this study was 1986–2006. The reported data include all suspected cases of acute meningitis, according to the standard clinical diagnosis by the WHO (1998): a sudden onset of fever (> 38.5°C rectal or 38.0°C axillary) and one or more of the following signs: stiff neck, altered consciousness, or other meningeal signs. In patients < 1 year of age, a suspected case occurs when fever is accompanied by a bulging fontanelle. Suspected cases may include meningitis caused by *Streptococcus pneumonia* and *Haemophius influenzae* b. However, *N. meningitidis* is the only pathogen associated with epidemics of meningitis. Average population density per district was calculated by dividing the district population in each year by the district area.

We modeled the seasonal number of cases (counts), which we defined as cases reported from January through May (the meningitis season). Weekly data for this period were aggregated at both national and district levels for each year. We examined whether climate conditions before January, including dust concentration, could be used to predict the meningitis incidence during January through May. There is a lack of historical and spatially resolved data on predictors related to population immunity, such as carriage prevalence, seroprevalence, vaccination coverage, and introduction of new clones. Therefore, we used the early incidence (i.e., cases per 100,000 population diagnosed in December) as a proxy measure of population carriage and/or susceptibility. de Chabalier et al. (2000) reported that major epidemics in Niger often showed higher incidence early in the season than minor epidemics.

*Climate and dust model data*. The atmospheric and dust data used in this study were derived using the recently developed online regional atmospheric dust model NMMB/BSC-Dust (Pérez et al. 2011), which simulates soil dust aerosol emission, transport within the atmosphere, and deposition. The resolution of the model was set to 1° × 1° and the simulation covered 1986–2006, coincident with the MDSC epidemiological data. The simulation was initialized every 24 hr, and the boundary conditions were taken from the global National Centers for Environmental Prediction Reanalysis-I (Kistler et al. 2001) for pressure level data, and from the Global Land Data Assimilation System II database (Rodell at al. 2004) for soil moisture and temperature. We used surface dust concentration estimates from a model given the paucity of direct *in situ* measurements especially at district level. The soil dust aerosol component of the model was thoroughly evaluated with existing satellite and *in situ* data over the region of interest (Ceccato et al. 2013; Pérez et al. 2011), showing daily aerosol optical depth correlations around 0.6 (*p* < 0.05). Additional details on the dust model and its suitability for the present study are provided in the Supplemental Material (“NMMB/BSC-Dust model”).

We considered model variables that characterize the Harmattan: zonal wind [i.e., the component of the horizontal wind toward east (meters per second)], meridional wind [i.e., the component of the horizontal wind toward north (meters per second)], wind speed (meters per second), humidity [specific (kilograms per cubic meter), absolute (kilograms per kilogram), and relative (percent)], temperature (kelvin), and surface dust concentration (micrograms per cubic meter). For wind, humidity, and temperature we used outputs at the pressure level of 925 hPa, which were consistent with values close to ground level in Niger. For dust concentration we used the particulate matter fraction ≤ 10 μm in size at 10 m above ground level.

For the national level, data were spatially averaged over the region 0.1° E to 14.2° E longitude and 12.3° N to 17.3° N latitude, which encompasses southern Niger. The northern region of Niger was excluded from this analysis because this region is scarcely populated due to the presence of the Sahara desert, and few meningitis cases occur.

We considered climate variables averaged over a range of consecutive months from September through December to explore whether climate and dust conditions leading up to January (before alert and epidemic thresholds are typically crossed) could be used to predict the extent of the following meningitis season. We applied a natural log (ln)–transform to the climate and dust variables, so they were approximately normally distributed. Because the monthly-averaged zonal and meridional wind components over the period and region of interest were negative (the Harmattan blows from north to south and from east to west), the absolute value of these variables was applied before the ln-transform. For inclusion in our models, we considered the available ln-transformed climate/dust variables with the largest Pearson correlation coefficients with the ln-transformed national seasonal meningitis count data: temperature from September through December at 925 hPa (*T_SDt_*), average zonal wind from November through December at 925 hPa (*U_NDt_*), average meridional wind from November through December at 925 hPa (*V_NDt_*), average wind speed at 925 hPa from November through December (*UV_NDt_*) and in December (*UV_Dt_*), and average dust concentration from September through December (*Dust_SDt_*) and from October through December (*Dust_ODt_*). The largest correlation value had the greatest magnitude of all the statistically significant (*p* < 0.05) correlation coefficients calculated for each climate variable. Interestingly, humidity before January did not show a significant correlation with the seasonal incidence.

*Modeling approach and evaluation criteria*. Under the assumption that the meningitis count data were overdispersed, we initially assumed that *Y_t_*, the number of cases observed in the January–May period of year *t* (where *t* = 1986…2006), followed a negative binomial distribution, with mean parameter μ*_t_,* overdispersion parameter θ, and variance σ*_t_* = (μ*_t_* + μ*_t_*^2^)/θ*_t_* (Lawless 1987). We determined the linear combination of risk factors that best represented the variability in the mean meningitis counts on the ln scale, ln(μ*_t_*). Maximum likelihood techniques were used to fit each of the models using the R statistical package (version 2.14.1; http://www.r-project.org/).

*National-level models*. At the national level, we modeled the seasonal counts *Y_t_* using a negative binomial distribution, and considered three different approaches to model the mean, μ*_t_.* First, we tested to what extent meningitis incidence before the onset of the disease in January could explain the seasonal incidence during January through May:

ln(μ*_t_*) = α + β*E_t_ +* ln(*N_t_*), [1]

where *E_t_* is the ln-transformed December incidence and *N_t_* is the national population count in meningitis-year *t.* The second model was specified as


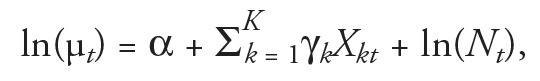
 [2]

where *X_kt_* is the *k* selected ln-transformed climate and dust variables. Finally, we used both climate/dust variables and December incidence as


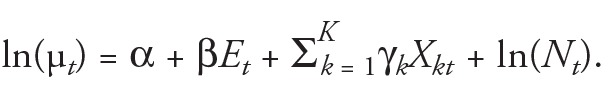
 [3]

A key concept of our model building and model selection was parsimony: Our goal was to build and select a model that was as simple as possible, while still explaining a significant amount of variability in the data. We used backward-selection to determine which of the climate/dust variables to include in the final national-level models. All of the shortlisted variables were included in an initial full model, and variables that did not explain a sufficient amount of variability in the data were removed from the model one at a time. We used likelihood ratio tests (Vuong 1989) to determine whether a variable’s contribution was sufficient or not (*p* < 0.05), in addition to testing for the significance of the overdispersion parameter. The final model was limited to variables that were significant predictors of seasonal meningitis incidence.

To evaluate the performance of each of the models, we used comparison measures and goodness-of-fit statistics including the Akaike information criterion (AIC), the pseudo-*R*^2^, and the Pearson correlation between the observed data and the resulting cross-validated predictions on the ln-incidence scale (CVC). CVC estimates were derived by fitting the model to the data with 1 year excluded and using the fitted parameter estimates to predict the excluded data. We used the “deviance-based” pseudo-*R*^2^ (Mittlböck and Waldhör 2000), which is restricted to the interval [0,1] and is interpreted as the amount of variability explained by the model.

We also analyzed each model’s ability to detect whether or not a particular incidence-based threshold had been exceeded, such that if the fitted probability of *y_t_* exceeding a threshold *K* was greater than some value *c* (where 0 *< c <* 1), then we predict that *y_t_ > K.* To account for the case where a small number of years had a large influence on the fit of the models, we used the cross-validated predicted values ^^~^^*y_t_* as opposed to the fitted time series ^^^^^*y_t_* to calculate the exceedance probabilities. We assumed a threshold *K* of 100 per 100,000 population (de Chabalier et al. 2000), and for a sequence of values for *c*—that is, 51 evenly spaced values between 0 and 1 (0.02 increments)—we calculated the sensitivity [SENS = true positives/(true positives + false negatives)], specificity [SPEC = true negatives/(true negatives + false positives)], and the scaled Hanssen and Kuipers score [HKS = (SENS + SPEC)/2, where HKS = 1 when the model generates perfect predictions, and HKS = 0.5 when the model performs no better than random]. The results presented are those corresponding to the value of *c* that minimized the equation (1-SENS)^2^ + (1-SPEC)^2^—that is, the value of *c* that simultaneously maximized the SENS and SPEC of the model estimates.

*District-level models*. At the district level, we analyzed whether the estimated effects of climate/dust and early incidence observed at the national level persisted at the district level, and whether the size of the estimated effect differed from that observed at the national level. For climate/dust, we considered both large-scale (national-level) covariates and local (district-level) deviations from the large-scale covariates—the difference between the district level and national level in the models under consideration.

We considered three model categories (based on early incidence, climate/dust, and both). For each category, we applied the corresponding selected national-level model to the district-level count data, and we considered two additional models including district-level covariates to assess the influence of including district-level information: one model with a universal intercept α, and one with a district-specific intercept α*_i_* (for each district *i*) to account for unexplained district differences. We used likelihood ratio tests to determine which covariates to include in the models including district-level covariates. In these models, we considered national-level ln-transformed climate/dust covariates (*X_kt_*), district-level climate/dust deviations from the national average (Δ*x_kit_*) (Δ*x_kit_* = *x_kit_* – *X_kt_*, where *x_kit_* is the district-level ln-transformed climate/dust variable), and ln-transformed early incidence in December at the national level (*E_t_*) and at the district-level (*e_it_*). As additional between-district variability is to be expected (since the spatiotemporal variability of climate in the region is lower than that of meningitis) we considered additional covariates that may explain part of the spatial variability including population density (*d_it_*), whether the district was classed as urban (*1_Ui_*) or rural (*1_Ri_*) (where *1_Ui_* = 1 and *1_Ri_* = 0 if the district is classified as urban, and *1_Ui_* = 0 and *1_Ri_* = 1 if the district is classified as rural), and the longitude (*lon_i_*) and latitude (*lat_i_*) of the centroid of the district. We also included the ln-transformed average early incidence of meningitis in December over all districts adjacent to each district (referred to as neighboring districts) (*^^–^^e_it_*) and the ln-transformed population size (*N_it_*) as an offset.

With regard to the climate/dust covariates, if one of the national level or the district-level deviation from the national average was significant, we retained both in the model. Three districts were classified as urban: Maradi, Niamey, and Zinder.

We also evaluated the performance of the district-level models with respect to detecting whether a particular incidence threshold *K* was exceeded using similar methods to those described for the national-level models, with the additional calculation of positive predictive value [PPV = true positives/(true positives + false positives)] and negative predictive value [NPV = true negatives/(true negatives + false negatives)]. We initially used a threshold of 100 per 100,000, and we then explored the dependence of the results on both the incidence threshold *K* and the method of selecting the cutoff value *c*.

## Results

*National level*. Summary data for the national-level models are presented in Table 1. In applying the stepwise backward model selection process, the final climate-only and climate-plus-early-incidence models contained a single climate variable, namely the average zonal wind during November through December (*U_NDt_*). The climate-plus-early-incidence model had pseudo-*R*^2^ = 0.57 and CVC = 0.59. The fit of the model where the single climate variable was the average wind speed during November through December (*UV_NDt_*) or the average dust concentration during October through December (*Dust_ODt_*) was statistically indistinguishable from the model that included *U_NDt_*, consistent with the high correlations among these variables (Pearson correlation coefficients 0.84–0.86). In contrast to results of Yaka et al. (2008), the meridional wind component (*V_NDt_*) model resulted in inferior scores.

**Table 1:** Model comparison and goodness-of-fit summaries for national-level negative binomial models fitted to the ln-incidence count data over the period 1987–2006.

Model	AIC	Pseudo-*R*^2^	CVC	SENS	SPEC	HKS
*E*_*t*_	395	0.24	0.38	0.40	1.00	0.70
*U*_*NDt*_^*a*^	387	0.49	0.51	1.00	0.60	0.80
*U*_*NDt*_ + *E*_*t*_^*a*^	385	0.57	0.59	0.80	0.87	0.83
*UV*_*NDt*_	388	0.47	0.51	1.00	0.53	0.77
*UV*_*NDt*_ + *E*_*t*_	385	0.57	0.60	0.80	0.80	0.80
*Dust*_*ODt*_	388	0.47	0.46	1.00	0.60	0.80
*Dust*_*ODt*_ + *E*_*t*_	386	0.55	0.56	0.80	0.93	0.87
*V*_*NDt*_	394	0.29	0.34	0.60	0.53	0.57
*V*_*NDt*_ + *E*_*t*_	392	0.42	0.48	0.60	0.87	0.73
Abbreviations: AIC, Akaike’s information criterion; CVC, Pearson correlation between the observed data and the resulting cross-validated predictions on the ln-incidence scale; *Dust*_*ODt*_, ln-transformed average dust concentration during October–December (μg/m^3^); *E*_*t*_*,* ln-transformed early incidence in December; HKS, scaled Hanssen and Kuipers score; SENS, sensitivity; SPEC, specificity; *U*_*NDt*_, *V*_*NDt*_, and *UV*_*NDt*_*,* ln-transformed average values during November–December for zonal wind (m/sec), meridional wind (m/sec), and wind speed (m/sec) at 925 hPA. ^***a***^Indicates the models selected during the model selection process. SENS, SPEC, and HKS were calculated using a threshold of 100 cases per 100,000.						

Models that incorporated both climate/dust and early incidence were superior in fit to both the early-incidence-only model, and the climate-only models (Table 1). Climate and dust had a greater impact on model fit than early incidence, as shown by the increases in pseudo-*R*^2^ and CVC, and decreases in AIC, between the climate-plus-early-incidence models and the corresponding models with early incidence or the climate component only.

**Figure 1:**
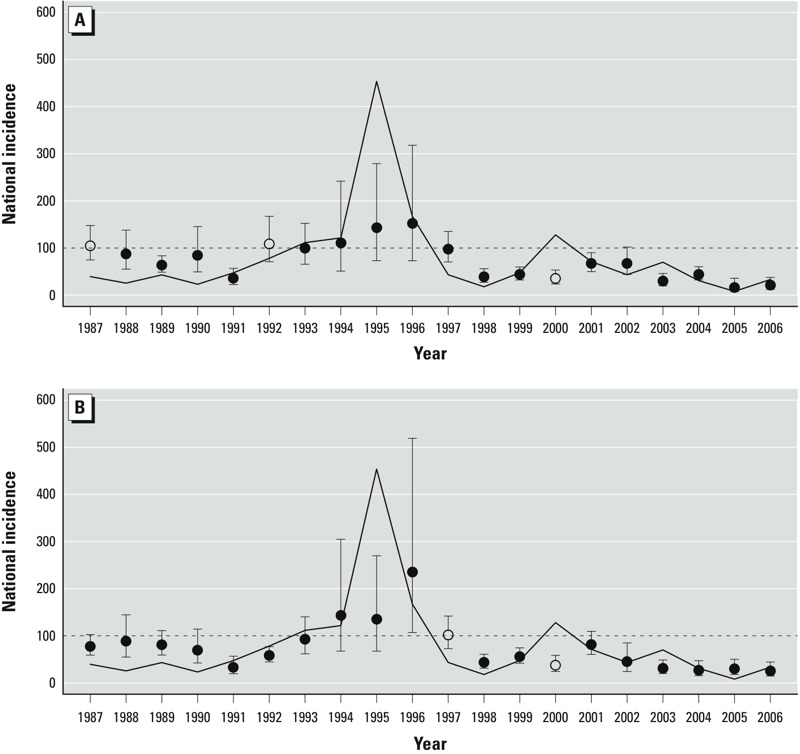
Observed national incidence (solid black line) and cross-validated national incidence predictions (circles), plus 95% CIs obtained by fitting a negative binomial model to the national count data, using November–December zonal wind (925 hPa) and December incidence as predictors (*A*), and using October–December dust concentration and December incidence as predictors (*B*). Black circles denote those predictions that were correctly assigned to be either above or below 100 cases per 100,000, whereas open circles are incorrect predictions. Decision cutoff values *c* of 0.42 (*A*) and 0.36 (*B*) were used.

Figure 1 presents the cross-validated results of the model with both average zonal wind during November through December (*U_NDt_*) and early incidence (*E_t_*) as covariates. Using a probability decision cutoff *c* of 0.42 and cross-validated predictions ^^~^^*y_t_*, 4 of 5 years were correctly predicted to have exceeded 100 cases per 100,000 over the 20-year period, whereas 2 of the remaining 15 years were incorrectly predicted to exceed it (SENS = 0.80, SPEC = 0.87, HKS = 0.83). The model captured the maximum incidence in 1995, although the magnitude was underestimated. Figure 1 also includes the results of the model with average dust concentration during October through December (*Dust_ODt_*) and early incidence as covariates. Using a probability decision cutoff of 0.36 and cross-validated predictions ^^~^^*y_t_*, 4 of 5 years were correctly predicted to have exceeded the threshold, whereas 1 of the remaining 15 years was incorrectly predicted to exceed it (SENS = 0.80, SPEC = 0.93, HKS = 0.87).

*District level*. Table 2 presents the model comparison and goodness-of-fit statistics for the district-level models. The climate/dust variables selected for inclusion in the models were the average zonal wind (November–December) at the national level (*U_NDt_*), the district-level deviation of the zonal wind from the national-level average (Δ*U_NDit_*), the average wind speed (November–December) at the national level (*UV_NDt_*), the district-level deviation of the wind speed from national-level average (Δ*UV_NDit_*), the average dust concentration (October–December) at the national level (*Dust_ODt_*), and the district-level deviation of the dust concentration from the national-level average (Δ*dust_ODit_*). The inclusion of both national and district-level covariates (Table 2, models 2, 5, and 8), as opposed to using national-level only (models 1, 4, and 7), resulted in a statistically significant (*p* < 0.05) improvement in the fit of the model. Although the inclusion of district-specific observed covariates such as population density and latitude explained some of the between-district variability, there were still unexplained differences between the districts, as indicated by the significant improvements made in the fit of the models when a district-specific intercept (α*_i_*) is included (models 3, 6, and 9). Similar to the national-level results, both early incidence (national, district, and averaged over neighbors) and wind and dust information (national and district) contributed to the fit of the model, with wind and dust information having the greater influence.

**Table 2:** Model comparison and goodness-of-fit summaries of the district-level negative binomial models fitted to the ln-incidence count data over the period 1987–2006.

Model	Covariates	AIC	pseudo-*R*^*2*^	CVC
1	*E*_*t*_	8,594	0.10	0.17
2	*E*_*t*_, *e*_*it*_, *ē*_*it*_, *I*_*Ui*_, *I*_*Ri*_, *d*_*it*_, *lat*_*i*_	8,478	0.21	0.43
3	*E*_*t*_, *e*_*it*_, *ē*_*it*_, α_*i*_	8,478	0.24	0.42
4	*U*_*NDt*_	8,531	0.16	0.25
5	*U*_*NDt*_, *ΔU*_*NDit*_, *Dust*_*ODt*_, *Δdust*_*ODit*_, *d*_*it*_, *lat*_*i*_	8,421	0.26	0.41
6	*U*_*NDt*_, *ΔU*_*NDit*_, *Dust*_*ODt*_, *Δdust*_*ODit*_, *d*_*it*_, α_*i*_	8,369	0.34	0.44
7	*E*_*t*_, *U*_*NDt*_	8,499	0.19	0.28
8	*E*_*t*_, *e*_*it*_, *ē*_*it*_, *U*_*NDt*_, *ΔU*_*NDit*_, *UV*_*NDt*_, *ΔUV*_*NDit*_, *Dust*_*ODt*_,*Δdust*_*ODit*_, *d*_*it*_, *lat*_*i*_	8,303	0.36	0.52
9	*E*_*t*_, *e*_*it*_, *ē*_*it*_, *U*_*NDt*_, *ΔU*_*NDit*_, *Dust*_*ODt*_, *Δdust*_*ODit*_, *d*_*it*_, α_*i*_	8,275	0.41	0.55
9N	*E*_*t*_, *U*_*NDt*_, *Dust*_*ODt*_, *d*_*it*_, α_*i*_	7,994	0.32	0.46
9D	*e*_*it*_, *ē*_*it*_, *U*_*NDit*_, *dust*_*ODit*_, *d*_*it*_, α_*i*_	7,898	0.40	0.56
Abbreviations: α_*i*_ district-specific intercept; AIC, Akaike’s information criterion; CVC, Pearson correlation between the observed data and the resulting cross-validated predictions on the ln-incidence scale; *d*_*it*_, district population density*; Dust*_*ODt*_, ln-transformed average dust concentration at national level during October–December (μg/m^3^); *dust*_*ODit*_, ln-transformed average dust concentration at district level during October–December (μg/m^3^); *E*_*t*_, *e*_*it*_ and *ē*_*it*_, ln-transformed early incidence in December at national level, district level, and averaged over neighboring districts (cases per 100,000); *I*_*Ui*_ and *I*_*Ri*_, urban and rural district indicators*; lat*_*i*_, district latitude; *U*_*NDt*_ and *UV*_*NDt*_, ln-transformed average values at national level during November–December for zonal wind (m/sec) and wind speed (m/sec) at 925 hPa; *ΔU*_*NDit*_, *ΔUV*_*NDit*_, and *Δdust*_*ODit*_, differences in the ln-transformed district-level zonal wind (m/sec), wind speed (m/sec), and dust concentration (μg/m^3^) compared with the ln-transformed national-level averages; *U*_*NDt*_, ln-transformed average zonal wind (m/sec) values at district level during November–December. Models 1–3 are based on early incidence. Models 4–6 are based on climate/dust covariates. Models 7–9 are based on early incidence and climate/dust covariates. Models 9N and 9D are similar to model 9 but with national-level covariates only (model 9N) and with district-level covariates only (model 9D) (both 9N and 9D also include district-specific intercepts).				

Table 3 presents SENS, SPEC, HKS, PPV, and NPV for each of the models with respect to an incidence threshold of 100 cases per 100,000. The best model with respect to these criteria and AIC, pseudo-*R*^2^, and CVC (Table 2) is model 9, which includes early incidence (national, district-level, and average of neighbors) and climate (national and district-level deviations from the national-level zonal wind and dust concentration), population density, and a district-specific intercept. However, this model represented only a small improvement over a model where the district-specific intercept was replaced by latitude and November–December wind speed (national level and the district-level deviation from the national level) (Table 2, model 8).

**Table 3:** Threshold-based results obtained for a range of district-level models produced using a threshold of 100 cases per 100,000 and using both sensitivity (SENS) and specificity (SPEC) to select the cutoff value *c*.

Model^*a*^	SENS	SPEC	HKS	PPV	NPV
1	0.5970	0.6451	0.6211	0.2778	0.8750
2	0.6119	0.6843	0.6481	0.3071	0.8852
3	0.5448	0.7457	0.6423	0.3288	0.8775
4	0.7015	0.5461	0.6238	0.2611	0.8889
5	0.7164	0.5973	0.6569	0.2892	0.9021
6	0.7090	0.6263	0.6677	0.3025	0.9039
7	0.6045	0.7082	0.6564	0.3214	0.8868
8	0.6493	0.7287	0.6890	0.3537	0.9008
9	0.6791	0.7218	0.7005	0.3583	0.9077
^***a***^Model numbers correspond to the models listed in Table 2.					

Table 4 includes the estimated coefficients for model 9. Both the national-level and district-level deviation coefficients for zonal wind and dust concentration are statistically significant, indicating that the national and district-level data make independent contributions to the fit of the model. A positive relationship was observed between zonal wind and meningitis incidence, indicating that stronger winds from an easterly direction were followed by an increase in cases. Despite the negative coefficient multiplying the district-level deviation dust covariate, the overall effect of dust [2.09 × *Dust_ODt_* – 1.36 × (*dust_ODit_* – *Dust_ODt_*)] is never to reduce meningitis incidence because the district-level dust concentration is never greater than roughly twice the national average (note that we refer to non–ln-transformed values). This negative coefficient means that dustier districts have lower incidence in this model. However, the incidence variations between districts due to dust is small compared with the estimated effect of the national-level dust concentration. The negative coefficient may be an artifact of uncertainties in district-level dust variations that are supplied by our climate model in the absence of direct measurements.

**Table 4:** Estimated coefficients (95% CIs) and p-values obtained by fitting model 9, model 9N, and model 9D.

Variable^*a*^	Model 9		Model 9N		Model 9D	
	Estimate (95% CI)	*p*-Value	Estimate (95% CI)	*p*-Value	Estimate (95% CI)	*p*-Value
*U*_*NDt*_	1.94 (0.79, 3.11)	0.0003	2.40 (1.19, 3.62)	< 0.0001
*ΔU*_*NDit*_	4.31 (2.94, 5.67)	< 0.0001
*U*_*NDit*_					3.19 (2.44, 3.93)	< 0.0001
*Dust*_*ODt*_	2.09 (1.18, 2.98)	< 0.0001	1.26 (0.34, 2.16)	0.0016
*Δdust*_*ODit*_	–1.36 (–2.48,–0.27)	0.0051
*dust*_*ODit*_					0.51 (–0.02, 1.02)	0.0298
*E*_*t*_	–0.18 (–0.37, 0.01)	0.0330	0.40 (0.23, 0.57)	< 0.0001
*e*_*it*_	0.23 (0.13, 0.32)	< 0.0001			0.21 (0.12, 0.32)	< 0.0001
*ē*_*it*_	0.38 (0.24, 0.51)	< 0.0001			0.33 (0.21, 0.45)	< 0.0001
d_it_	2.00 (1.43, 2.57)	< 0.0001	1.54 (0.95, 2.13)	< 0.0001	2.05 (1.46, 2.62)	< 0.0001
Models 9N and 9D are similar to model 9 but with national-level covariates only (model 9N) and with district-level covariates only (model 9D) (both 9N and 9D also include district-specific intercepts).^***a***^Variables are described in Table 2.						

Model 9 had the unusual feature that district-level seasonal incidence decreased as national-level early incidence increases. (The coefficient of *E_t_* in Table 4 is negative.) However, the coefficient is not statistically distinct from zero, and its influence is small in practice, compared with the estimated effect of early incidence in the district (*e_it_*) and the immediate neighbors (*^^–^^e_it_*). The negative value of *E_t_* may be partly an artifact of our simple model, which does not distinguish between early incidence within remote districts and districts that are nearby but not neighboring (and whose influence is not included within *^^–^^e_it_*). The use of the national average to represent the influence of both these districts may be too restrictive, resulting in the counterintuitive negative (but small) estimated effect of *E_t_*.

In Table 2 and Table 4 we compared the results of model 9 with model 9N, based upon national-level covariates only, and model 9D, constructed from district-level covariates only. (All models include district-specific intercepts.) These additional models show the expected increase of seasonal incidence with increasing wind, dust, early incidence, and population density. In contrast to model 9, early incidence at the national level is associated positively with meningitis incidence when district-level deviation covariates are not included (model 9N). In model 9D, the removal of national-level early incidence only slightly weakened the influence of early incidence in adjacent districts. The comparison of goodness-of-fit among models 9, 9N, and 9D in Table 2 demonstrates that the inclusion of district-level variables provides a better fit and that the addition of the national-level data does little to improve predictions at district-level.

**Figure 2:**
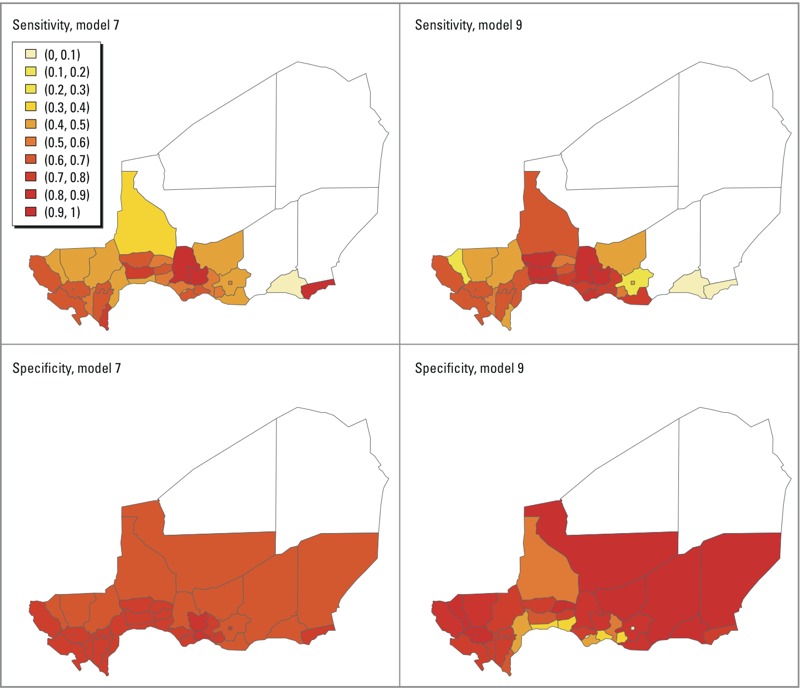
Maps of sensitivity and specificity based on predictions from a model that includes zonal wind and December incidence at national level (model 7, as defined in Table 2) (left) and a model with zonal wind, dust, and December incidence at the national and district levels, average December incidence of neighboring districts, population density, and a district-specific intercept (model 9, as defined in Table 2) (right). A threshold of 100 cases per 100,000 was used, and the value of c was selected as the value that simultaneously optimized both sensitivity and specificity.

Figure 2 shows district-level sensitivity and specificity estimates for model 7 (which includes early incidence and zonal wind at the national level only) and model 9. For both models, sensitivity was rather heterogeneous across the country, with model predictions for the central southern districts close to the border with Nigeria, where population density is highest, showing the highest sensitivity. District-level specificity was more homogeneous across the country, with model 9 estimates showing greater specificity than model 7 estimates, except in some of the southern districts.

**Figure 3:**
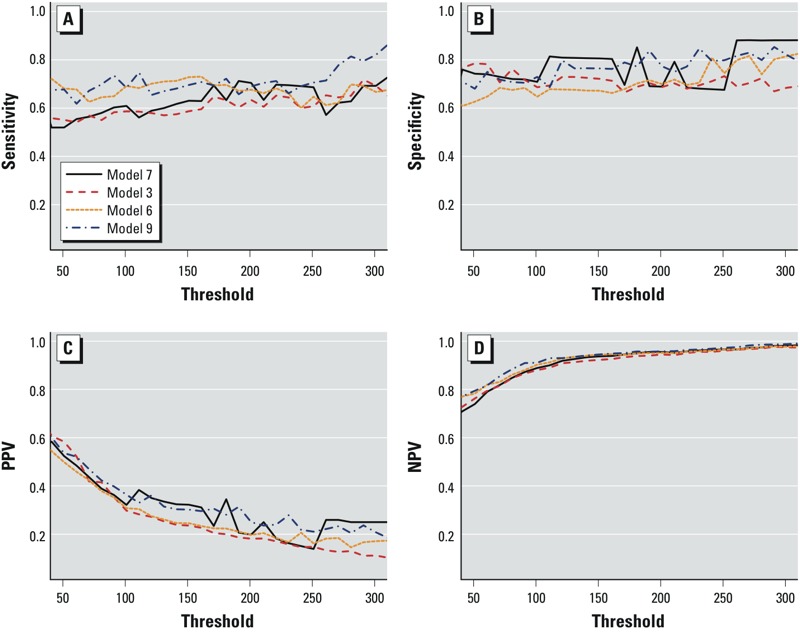
Plot of sensitivity (*A*), specificity (*B*), positive predictive value (PPV; *C*), and negative predictive value (NPV; *D*) against the epidemic incidence threshold (in cases per 100,000) for models 7, 3, 6, and 9. (See Table 2 for variables included in the numbered models.)

Threshold-based model evaluations are highly dependent on the selected threshold and the method to select the cutoff value *c.* Typically the threshold under consideration is policy driven—for example, there may be a particular threshold from which certain actions are initiated. For seasonal meningitis incidence there is no universally recognized threshold to categorize the season to be either “normal” or “high.” In the above analysis we used a threshold of 100 cases per 100,000 (de Chabalier et al. 2000). We additionally explored the dependence of our results on this threshold using a range between 50 and 300 cases per 100,000 for which 35% and 5% of district-seasons crossed the threshold, respectively. Figure 3 presents plots of SENS, SPEC, PPV, and NPV for four different models over this range. Models considered included a model with early incidence and zonal wind at 925 hPa at the national level only (model 7); a model with both national and district-level early incidence, average early incidence averaged over neighboring districts, and a district-specific intercept (model 3); a model with national and district-level zonal wind and dust concentrations and a district-specific intercept (model 6); and a model with both national- and district-level early incidence, national- and district-level zonal wind and dust concentrations, average early incidence in neighboring districts, population density, and a district-specific intercept (model 9). Models 6 and 9 generally outperformed the other models with regard to sensitivity (around 0.7 for thresholds between 50 and 170 per 100,000), PPV, and NPV. With respect to specificity, none of the models considered consistently outperformed the other three. As expected, PPV decreased and NPV increased as the threshold increased because, as the number of epidemics decreased, the number of false positive and true negative predictions increased while true positive and false negative predictions decreased.

When determining how the cutoff value *c* is selected, the relative importance of sensitivity, specificity, PPV, and NPV needs to be considered with respect to some form of cost–benefit measure: For example, is it feasible to vaccinate a large number of people unnecessarily if it means that a large proportion of cases are prevented? We performed additional analyses in which simultaneous optimization of sensitivity, specificity, PPV, and NPV was the criterion used to select *c* [i.e., the value of *c* that minimized the equation (1-SENS)^2^ + (1-SPEC)^2^ + (1-PPV)^2^ + (1-NPV)^2^], instead of using optimization of sensitivity and specificity only (Table 5). The method had no influence on results obtained for model 3. For results obtained for model 6 and model 9, we observed that generally NPV was not essentially affected by the optimization technique, specificity was greater using the alternative optimization technique, whereas sensitivity and PPV decreased and increased, respectively, by approximately equal amounts. These results highlight the need to prioritize these parameters for optimization according to the specific public health context when developing the model.

**Table 5:** Threshold-based summaries obtained using a threshold of 100 cases per 100,000 and using either SENS and SPEC only or SENS, SPEC, PPV, and NPV to optimize the decision cutoff *c*.

Model	Optimization criteria	SENS	SPEC	HKS	PPV	NPV
3	SENS, SPEC	0.5448	0.7457	0.6453	0.3288	0.8775
3	SENS, SPEC, PPV, NPV	0.5448	0.7457	0.6453	0.3288	0.8775
6	SENS, SPEC	0.7090	0.6263	0.6677	0.3025	0.9039
6	SENS, SPEC, PPV, NPV	0.3433	0.9283	0.6358	0.5227	0.8608
9	SENS, SPEC	0.6791	0.7218	0.7005	0.3583	0.9077
9	SENS, SPEC, PPV, NPV	0.5672	0.8584	0.7128	0.4780	0.8966

## Discussion

At the national level, both the early incidence and November–December averaged zonal wind together provided the best fit (pseudo-*R*^2^ = 0.57), with the climate variable having a greater impact on the fit. The sensitivity and specificity of this national model to predict epidemics > 100 cases per 100,000 population were 0.8 and 0.87, respectively. A national model with October–December dust concentration and early incidence performed indistinguishably well (pseudo-*R*^2^ = 0.55, SENS = 0.80, SPEC = 0.93). Our results suggest a significant influence of early-season conditions on the initial slope and final amplitude of the incidence of meningitis during epidemics in the study area. Indeed, de Chabalier et al. (2000) showed that epidemics that occurred early in the meningitis season in Niger were characterized by a more rapid increase and higher seasonal peaks in the incidence of meningitis. In our study, wind and dust conditions during the period January–March did not correlate with seasonal incidence (data not shown). Therefore, with this approach, forecasting seasonal incidence (January–May) could be based on observed early-season climate/dust information (November–December) and would not rely upon uncertain seasonal climate forecasts.

At the district level, early-season zonal wind and dust (i.e., in November–December), along with the early incidence in December and the district population density, represented the spatiotemporal variability of the disease with pseudo-*R*^2^ = 0.41 and CVC = 0.55. The inclusion of zonal wind and dust information substantially increased our ability to predict which districts would exceed a particular incidence threshold, as it increased model sensitivity and/or PPV depending on optimization criteria. District-specific intercepts also improved model performance by accounting for between-district variability that was not explained by other model covariates.

The use of suspected cases instead of confirmed cases and the lack of historical vaccination data are limitations of current modeling and forecasting approaches, including our study. Our model uses early incidence as a proxy measure of population susceptibility and/or carriage prevalence. Although this may at least partially account for vaccination in preceding years, the incidence reported in a given district may have been affected by the reactive vaccination within the same season. Following the outbreaks of meningitis in 1995–1996 in West Africa, an International Coordinating Group on Vaccine Provision for Epidemic Meningitis Control (WHO, Geneva, Switzerland) was established in January 1997 to coordinate the best use of the limited amount of vaccine available and to ensure a better distribution of the meningitis vaccine. Therefore, the extent to which reactive vaccination affected the dynamics of the disease is uncertain. Future modeling studies may attempt to reconstruct immunity patterns (natural and vaccination-induced) from data on cases, population size, and climate seasonality and interannual variability. The use of more complex mechanistic models that account for the nonlinear interaction between climate and susceptibility, together with the availability of new data on carriage rates, vaccination coverage, and respiratory viral infections, is expected to enhance our understanding of the epidemics and eventually serve as more precise prediction tools.

The accuracy of our models to predict epidemics after the introduction of conjugate A vaccine in 2010 (LaForce et al. 2007) must be tested because of the possible near future elimination of large epidemics of serogroup A. However, meningitis is likely to continue to be a problem within the belt due to the length of time it will take to vaccinate the entire at-risk population and the potential emergence of other serogroups such as W135 and X. Further, a forecasting system based on pre-conjugate vaccination data could be used retrospectively to disentangle the confounding effect of climate in the assessment of the impact of the new vaccine on carriage and incidence.

## Conclusions

We demonstrated the potential and limitations of using early-season wind and soil dust information to predict meningitis epidemics in Niger based on data from 1986 to 2006. Precise predictions of epidemics cannot be based solely on climate data and coarse proxies of susceptibility. However, our model (if amended to account for the introduction of conjugate A vaccine) could lead to an early-season alert that climate and other conditions are potentially conducive to an epidemic, which could initiate an early response strategy including increased surveillance, ensuring that stocks of vaccines are in-country, that protocols and procedures are in place, and that district health teams and members of the public likely to be affected are forewarned and prepared. If the presence of the pathogen or an increase in incidence is subsequently confirmed based on surveillance systems at district or finer levels (Paireau et al. 2012; Tall et al. 2012), early warnings could be followed by additional actions as needed.

## Supplemental Material

(107 KB) PDF
