# Supplementary material for: Soil Dust Aerosols and Wind as Predictors of Seasonal Meningitis Incidence in Niger
Source: Environ Health Perspect. 2014 Mar 17;122(7):679–86. doi: 10.1289/ehp.1306640 (PMC4080544; doi:10.1289/ehp.1306640)
Supplement: (107 KB) PDF [file ehp.1306640.s001.pdf]

Environmental Health Perspectives doi:10.1289/ehp.1306640

Supplemental Material

Soil Dust Aerosols and Wind as Predictors of Seasonal Meningitis Incidence in Niger

Carlos Pérez García-Pando, Michelle C. Stanton, Peter J. Diggle, Sylwia Trzaska, Ron L. Miller, Jan P. Perlwitz, José M. Baldasano, Emilio Cuevas, Pietro Ceccato, Pascal Yaka, and Madeleine C. Thomson

The NMMB/BSC-Dust model

The NMMB/BSC Dust model was developed at the Barcelona Supercomputing Center in collaboration with the NASA Goddard Institute for Space Studies and the National Centers for Environmental Prediction (Pérez et al. 2011). Recently, the World Meteorological Organization (WMO) has chosen Spain's bid to host the first Regional Meteorological Center specialized on Atmospheric Sand and Dust Forecast and has appointed the consortium formed by the Spanish State Meteorological Agency (AEMET) and the Barcelona Supercomputing Center (BSC-CNS) to house the center and release operational forecasts of atmospheric mineral dust for Northern Africa, Middle East and Europe (WMO 2014; http://sds-was.aemet.es). The new center will run the NMMB/BSC-Dust model used in this paper and will provide operational forecasts for Northern Africa, Middle East and Europe. The NMMB/BSC-Dust model was thoroughly evaluated and showed daily aerosol optical depth (AOD) correlations in the Sahel of around 0.6 ($p < 0.05$) on average (Ceccato et al. 2013; Pérez et al. 2011) when the model is compared to observed AOD from Sun-photometers and satellites. For additional results, the reader is referred to the Second delivery Report of the EU MACC project Workpackage 3.1 (Cuevas et al. 2011) available at http://sds-was.aemet.es/projects-research/MACCOINTWP3.1SeconddeliveryReport_Final.pdf. The report shows detailed model evaluation from daily to monthly and interannual timescales and an initial exploratory analysis of the meningitis database.

References

Ceccato P, Trzaska S, Pérez García-Pando C, Kalashnikova O, del Corral J, Cousin R, et al. 2013. Improving decision-making activities for meningitis and malaria, Geocarto Int. doi: 10.1080/10106049.2013.827749.

Cuevas E, Pérez C, Baldasano JM, Camino C, Alonso-Pérez S, Basart S. 2011. Meningitis linked to mineral dust transport in the Sahel. EU MACC project Workpackage 3.1 Second Delivery Report. Available: http://sds-was.aemet.es/projects-research /MACCOINTWP3.1Second deliveryReport_Final.pdf [accessed 18 February 2014].

Pérez C, Haustein K, Janjic Z, Jorba O, Huneeus N, Baldasano JM, et al. 2011. Atmospheric dust modeling from meso to global scales with the online NMMB/BSC-Dust model - Part 1: Model description, annual simulations and evaluation. Atmos Chem Phys 11: 13001-13027. doi: 10.5194/acp-11-13001-2011.

WMO (World Meteorological Organization). 2014. Sand and Dust Storm Warning Advisory and Assessment System (SDS-WAS) for Northern Africa-Middle East-Europe (NA-ME-E) Homepage. Available: http://sds-was.aemet.es [accessed 18 February 2014].
